# Perfluorocarbon-based oxygen carriers: from physics to physiology

**DOI:** 10.1007/s00424-020-02482-2

**Published:** 2020-11-03

**Authors:** Johannes Jägers, Anna Wrobeln, Katja B. Ferenz

**Affiliations:** 1 University of Duisburg-Essen, Institute of Physiology, University Hospital Essen, Hufelandstraße 55, 45122 Essen, Germany; 2 CeNIDE (Center for Nanointegration Duisburg-Essen) University of Duisburg-Essen, Carl-Benz-Strasse 199, 47057 Duisburg, Germany

**Keywords:** Artificial oxygen carriers, Perfluorocarbon emulsion, Perfluorocarbon-based artificial oxygen carrier, CYP450 uncoupling, Reticuloendothelial system uptake, Perfluorocarbon excretion

## Abstract

Developing biocompatible, synthetic oxygen carriers is a consistently challenging task that researchers have been pursuing for decades. Perfluorocarbons (PFC) are fascinating compounds with a huge capacity to dissolve gases, where the respiratory gases are of special interest for current investigations. Although largely chemically and biologically inert, pure PFCs are not suitable for injection into the vascular system. Extensive research created stable PFC nano-emulsions that avoid (i) fast clearance from the blood and (ii) long organ retention time, which leads to undesired transient side effects. PFC-based oxygen carriers (PFOCs) show a variety of application fields, which are worthwhile to investigate. To understand the difficulties that challenge researchers in creating formulations for clinical applications, this review provides the physical background of PFCs’ properties and then illuminates the reasons for instabilities of PFC emulsions. By linking the unique properties of PFCs and PFOCs to physiology, it elaborates on the response, processing and dysregulation, which the body experiences through intravascular PFOCs. Thereby the reader will receive a scientific and easily comprehensible overview why PFOCs are precious tools for so many diverse application areas from cancer therapeutics to blood substitutes up to organ preservation and diving disease.

## Introduction

Humankind has pursued the idea of rejuvenation and blood substitution since ancient times. In 8 AD, Ovid versified his thoughts on the magician Medea who—with a complex mixture of plants, stones and sand she had collected for 9 nights and 9 days—successfully regenerated the blood of the doter Aeson who rejuvenated into a 40-year-old man [45]. The first effective inter-human blood transfusion was performed on September 1st 1818, by James Blundell. But as human blood has always been a very precious and finite good, Amberson and Rhode were among the first researchers mixing isolated red blood cells (RBCs) from cattle, cats, dogs or humans with Ringer’s solution to simulate human blood in the 1930s [1]. They focused on the main ability of blood: Oxygen (O_2_) transport via RBCs. It took until the 1960s for the first artificial oxygen carriers made of engineered haemoglobin or other synthetic compounds to replace RBCs in blood substitution. This promised the end of the subjection to donated human blood for transfusion [83]. However, the interest in synthetic blood substitutes only increased gradually until the HI-virus-contaminated-donor-blood-crisis arose in the 1980s and vigorously accelerated the developmental research on artificial blood substitutes [8]. The use of perfluorocarbons bears attractive advantages over blood: Unlimited availability, no transmittance of diseases and no dependence on blood types [23]. This review intends to introduce the reader to these astonishing materials, which offer so many more chances of application beyond ordinary blood substitution.

## The very beginning

The work of Leland C. Clark and Frank Gollan on fluorinated hydrocarbons (perfluorocarbons, PFC) laid the foundation to the idea to utilize the O_2_ carrying and conducting features of these liquids for clinical application. In their famous experiment in 1966, they made mice dive in fluorobutyltetrahydrofuran (FX-80), which was equilibrated with 100% O_2_ [9]. Within this experiment, they showed that anaesthetized mammals could breathe this fluid and maintain their respiration for 4 h. In the same publication, they describe their second setup, in which they placed mice under a reverse funnel. The wide side of this funnel was placed under the surface of liquid PFC, which was equilibrated with 100% O_2_ so that the only O_2_ these mice were breathing emerged from the PFC. The mice showed no sign of hypoxia for hours, whereas mice in the same setup with water instead of the PFC died after a few minutes [9]. The latter experiment showed that PFCs are optimal gas transporters as they are not only able to store O_2_ but also to release enough of it to maintain mammalian life. These key properties gave rise to the idea of using these kinds of chemicals as artificial oxygen carriers in blood substitutes resulting in the first clinical use of a PFC-based artificial oxygen carrier named Fluosol-DA in 1980 [38].

## Perfluorocarbons

**Fig. 1:**
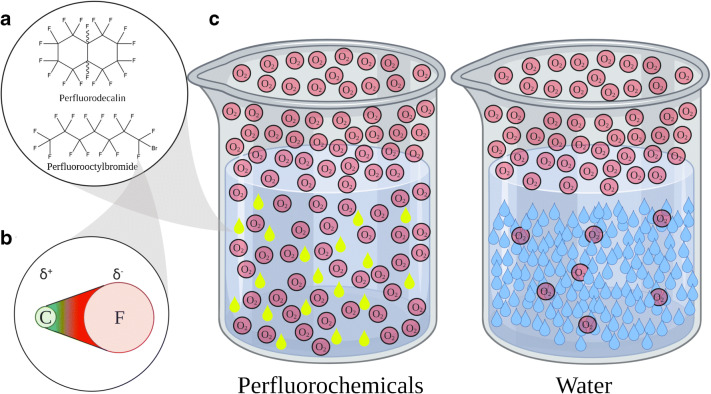
(A) PFD and PFOB are the most commonly used PFCs as oxygen carriers. (B) The carbon-fluorine-bond is extremely polarized; the probability of the presence of the electron is on the fluorine side. (C) Illustration of the O_2_ capacity of water and perfluorochemicals. At the same pO_2_, the amount of O_2_ dissolved in PFC is tremendously higher than the amount in water. Figure created with Biorender.com

PFCs are hydrocarbons whose hydrogen atoms are substituted either completely by fluorine atoms and sometimes additional other halogens (Fig. 1A). This substitution changes the physical properties of these organic compounds radically. Fluorine is the halogen of the second period; its atom radius is only twice the one of hydrogen, while its weight is 20 times the one of hydrogen. Among all the elements, fluorine is the most eager to suck electrons from other atoms, which tightens its bonds along the whole molecular structure and stiffens the carbon backbone of these fluorinated compounds (Fig. 1B) [88]. The carbon-fluorine bond is very strong (~ 484 kJ/mol) and extremely polar (almost ionic). However, this does not result in water solubility because the intrinsic symmetry annuls the polarity of each C–F bond with the whole PFC molecule becoming nonpolar (Fig. 1A). The tight bonds cause a more bulky shape of the PFC molecule so it needs more space in the water, which increases the hydrating energy. This effect decreases the solubility in water, compared to the corresponding hydrocarbon derivative [14]. On the other hand, this extreme polarity inhibits the formation of induced dipoles, which would lead to van der Waals forces, necessary for solubility in lipids. Therefore, PFCs are one of those rare species that are both hydrophobic and lipophobic.

All PFCs are able to dissolve tremendous amounts of gases. The two most commonly used PFCs are perfluorooctylbromide (PFOB) and perfluorodecalin (PFD, Table 1). These two PFCs can dissolve 527 and 403 mL_O2_/L_PFC_ at 1 atm (1 bar, 713 mmHg), respectively. Carbon dioxide can be dissolved up to 4 times the amount of O_2_ [102]. In comparison, the solubility of O_2_ in water is about 9 to 10 mL_O2_/L_water_ and in blood around 200 mL_O2_/L_blood_ [86, 94]. At this point please be reminded that 1 L of water contains 55 mol, whereas 1 L of PFD contains only 4.2 mol. Therefore, the molecular ratio of dissolved O_2_ is 1_O2_:200_water_ in water, but 5_O2_:1_PFD_ in PFD, resulting in a 1000× increased molecular solubility for PFD compared to water (Fig. 1C). Major properties for these calculations are listed in Table 1.

**Table 1:** Comparison of the main physical properties of water, PFD, PFOB and DDFP [42, 94, 102]

	Water	Perfluorodecalin (PFD)	Perfluorooctylbromide (PFOB)	Dodecafluoropentane (DDFP)
Formula	H_2_O	C_10_F_18_	C_8_BrF_17_	C_5_F_12_
Molar mass	18 g/mol	462 g/mol	499 g/mol	288 g/mol
Density	0.997 g/cm^3^	1.946 g/cm^3^	1.89 g/cm^3^	1.63 g/cm^3^ (liquid, 25 °C), 0.012 g/cm^3^ (gas, 37 °C)
Molar density	55.4 mol/L	4.2 mol/L	3.8 mol/L	0.04166 mol/L (gas, 37 °C)
Oxygen solubility (25 °C)	6.3 mL_O2_/L_H2O_	403 mL_O2_/L_PFD_	527 mL_O2_/L_PFOB_	29,421 mL_O2_/L_DDFP_ (gas, 37 °C)
Molar oxygen solubility	0.11 mL_O2_/mol_H2O_	95.68 mL_O2_/mol_PFD_	139.14 mL_O2_/mol_PFOB_	7.06*10^5^ mL/mol_DDFP_ (gas, 37 °C)
Molar ratio (oxygen/solute)	0.005 mol_O2_/mol_H2O_	4.27 mol_O2_/mol_PFD_	6.21 mol_O2_/mol_PFOB_	31.52 mol_O2_/mol_DDFP_ (gas, 37 °C)

The low molecule density of PFCs, mentioned above, explains the high O_2_ capacity and conductivity, which Clark and Gollan tested in their experiment from 1966. Let us carry out a thought experiment and consider solvent molecules to be pebbles on a road that you walk on barefoot. You can pass the pebbles, but you need to take your time and energy to carefully step between them. The O_2_ passes the “PFD pebbles” substantially faster than the “water pebbles”, because the PFD molecules leave substantially more space so that the O_2_ can move freely between the solvent molecules and pass them (Fig. 1C). The O_2_ capacity obeys Henry’s law, which means it is theoretically infinite assuming an infinite pO_2_.

## PFC-based oxygen carriers: emulsions, formation and storage

Less than a year later than their mouse-diving experiment, Clark and Gollan kept a rat Langendorff-heart beating by perfusing it with pure PFC [32]. In 1967, Sloviter annotated in his study: “Water and polar substances such as glucose and salts are virtually insoluble (in PFCs)” [87]. He was the first to emulsify (emulsifier: bovine serum albumin (BSA)) PFC in water and successfully perfuse isolated rat brains [87]. This emulsification paved the way to using PFCs as oxygen carriers in physiological systems by developing a way to allow the addition of, e.g., water-soluble nutrients and pharmaceuticals.

Emulsification is the process of mixing two or more immiscible liquids via building microbubbles. It generates thermodynamically metastable but kinetically stable mixtures. For deeper knowledge about the physics of emulsification, numerous reviews have been published [29, 93]. Briefly, the extreme hydrophobicity of PFCs makes the use of emulsifiers such as lipids, fluorinated compounds, tensides or proteins necessary to bypass the immiscibility with aqueous media, e.g. blood. Forming a microbubble is a thermodynamic process that is described by a variant of the Gibbs-Helmholtz-Equation: [28].

$$ \Delta G=\left(\gamma A\right)-\left(\varDelta S\ast T\right) $$

Δ*G* = cost of energy (Gibbs-enthalpy), *γ* = interfacial tension, *A* = interfacial area, Δ*S* = gain of disorder (entropy), *T* = energy (temperature)

The cost of energy (enthalpy) of a reaction must be negative to make the reaction proceeds spontaneously. As you can see in the equation, the interfacial area should be small to minimize the cost of energy. The lack of possible hydrogen bonds at the interface of the immiscible liquids causes interfacial tension, which increases the inner energy of the whole system. However, a few millilitres of an emulsion can contain an interfacial area the size of a football field or even larger. Emulsifiers adhere to the interface and represent a hydrogen bond acceptor that lowers the interfacial tension. This minimizes the unfavourable rise of energy caused by the increase of the interfacial area. Considering the second law of thermodynamics, systems tend to increase the intrinsic disorder. Solving an emulsifier in a solvent, e.g. water, decreases the disorder by forcing the water to form hydration spheres around the emulsifier. Adsorption at interfacial areas, therefore, increases the disorder of the system [27, 93]. As mentioned above, emulsions are metastable because the emulsion reaches the thermodynamic equilibrium only after a complete phase separation, and two major processes push this decay forward. The first one is coalescence. If you have ever observed raindrops running down a window, you might have seen them approaching each other, and as soon as the droplets’ surfaces touch, fusing into one big droplet. This event is coalescence and the driving force is the minimization of the interfacial area. The only effective way to avoid the fusion is to avoid the contact between the droplets. One way to accomplish this is to reduce Brownian movement of the droplet via cooling so that the droplets may not move too close to each other. A disadvantage of freezing an emulsion is that it may lead to decay [16, 30]. Another way to inhibit coalescence is to use charged emulsifiers like lipids or proteins to generate a high surface charge density, which leads to the repulsion of droplets [48, 77]. To envision this repulsion think of washing your hands with soap. The tensides in the soap give a dense negativity to your skin, which causes the two hands to repulse, leading to reduced friction, which feels slippery [40]. Furthermore, the emulsifier’s layer thickness, the emulsifier’s density at the interface and the droplet size contribute to the steric stabilization of an emulsion [46]. The repulsion between two droplets occurs at distances below twice the layer thickness. The thicker the layer, the earlier the repulsion occurs, which limits the possibility for the attractive forces (van der Waals forces) between two droplets to prevail at short distances. Additionally, the repulsive forces augment with increasing layer-thickness to droplet-size ratio [93]. Therefore, nano-emulsions, which show droplet diameters below 1 μm, are more stable than micro-emulsions, which exhibit droplet diameters larger than 1 μm. When the first Food and Drug Administration (FDA)–approved PFC emulsions such as Fluosol-DA and Perftoran came up in the 1980s, they suffered from flocculation, which gave the products weak shelf life [81]. The used emulsifiers were block polymers sometimes mixed with lecithin or phospholipids, which generate too little surface charge density to prevent this process [24]. Freezing the emulsion prior to storage was insufficient because of decay during thawing, which made it necessary to sonicate the emulsion prior to use. Even though these first-generation PFC-based oxygen carriers (PFOCs) showed major physiological side effects, the main reason for stopping the clinical trials in the 1990s was the insufficient shelf life [36, 51, 101]. Short time later, new PFC emulsions such as Oxycyte and Oxygent were developed using egg yolk lecithin as emulsifier and further block polymers (Proxanol 268), which allow for the formation of smaller droplets (50–100 nm) with a higher charge density [50, 62]. Those emulsions showed a higher stability, were heat sterilizable and could be stored frozen without decaying when thawing [23, 101]. Decay of these second-generation emulsions is the result of a second process: Ostwald ripening. Small droplets vanish in favour of the growth of bigger more stable ones. Again, the reduction of the interfacial area is the driving force, but in this case, diminishment by kinetic stabilization is impossible. The PFC inside the droplet evaporates or dissolves in the surrounding solvent until a bigger droplet captures it. Crucial factors herein are the solubility of the PFC in water and its vapour pressure, which forces the PFC to evaporate. Minimizing these factors by choosing the right PFC can minimize Ostwald ripening [43]. Recently developed PFC emulsions using PFD, which displays extremely low solubility in water, can reduce Ostwald ripening. In addition, the use of charged proteins like albumin as an emulsifier that forms a thick layer with a high surface charge density such as in A-AOCs helps to prevent flocculation and coalescence [17, 34].

## PFC-based oxygen carriers: droplets under attack

**Fig. 2:**
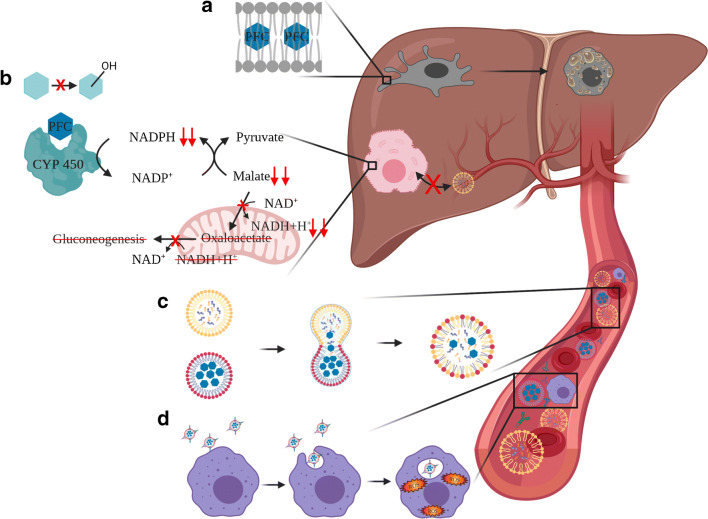
PFCs are inherently inert, but dependent on their lipo- and hydrophilicity and their vapour pressure they interact with the tissue and blood. (A) Some of the more lipophilic PFCs such as PFOB intercalate into the lipid bilayer of cell and organelle membranes [19]. The major part of PFCs are deposited inside the Kupffer cells which makes them appear foamy [84]. (B) PFCs can uncouple CYP450 monooxygenases, which reduces the detoxification capacity of the liver and causes differed glucose and lipid metabolism [21, 58, 71, 73, 85]. NAD/NADP: Nicotinamideadeninedinucleotide/-phosphate. (C) Proportional to the vapour pressure, phospholipid-wrapped PFCs (red) and natural lipid vesicles (yellow) inside the bloodstream may fuse and form hybrid vesicles [53, 99]. (D) PFC droplets are opsonized by complement factors or antibodies to be recognized and phagocytized by macrophages [52]. Figure created with Biorender.com

Entering the vascular system, PFC droplets face different obstacles, which they have to overcome to function as appropriate O_2_ shuttles. As mentioned above, size is an important factor influencing the stability of emulsions regarding shelf life but also the intravascular half-life [75]. The droplet size should be smaller than 0.5 μm mainly because of two reasons. On the one hand, the beneficial effect of the enhanced oxygen transport capacity decreases with increasing size of the droplet, because of reduced diffusivity, so that droplets with a size bigger than 0.5 μm need a treatment with 100% oxygen to work at their full potential [26]. On the other hand, droplets with a diameter over 0.5 μm may cause microembolism through obstruction of the microvascular system [104]. Both cause injury by oxygen toxicity and embolism, of the lung for example [80]. Therefore, especially the older formulations (Fluosol-DA and Perftoran) were associated with pneumonia. Additionally, the body is capable of discriminating endogenous and exogenous structures efficiently. Therefore, the body may confuse the droplets with vicious intruders such as microorganisms or tumour cells and might try to defend itself against this alleged danger. The frontline of this defence is the reticuloendothelial system (RES), which consists of several different cell types and proteins. The first step is that complement factors and antibodies recognize the PFC droplets and opsonize them. There are three ways to reduce this process: Firstly, through choosing the adequate emulsifiers such as endogenous proteins and lipids. Secondly, via the attachment of polyethylene glycol (so-called PEGylation) to the outside of the particle, which sterically hinders the general interaction of proteins with the droplet surface [25, 35, 52]. Thirdly, by carefully adjusting the droplet size, which influences the circulation of PFC droplets in the blood. In general, the smaller the droplet, the longer it will circulate in the bloodstream, with a minimum size of around 100 nm, below which the droplets will enter the endothelial cells via pinocytosis [15, 44]. By saying that, one has to admit that the RES might not be the only factor to explain the elimination from the vascular system. In addition, the fusion of PFCs wrapped in phospholipids with natural lipid vesicles inside the bloodstream may contribute to the particle clearance (Fig. 2C). The rate of this fusion of phospholipid-coated PFC droplets and, e.g., cholesterol vesicles or lipoproteins is proportional to the vapour pressure of the PFC used [53, 99]. Once the droplets are opsonized, circulating macrophages and monocytes take them up and transport them directly to the lung, where they can evaporate [95]. In addition, splenocytes and Kupffer cells also start phagocytosis and, thereby, store the incorporated PFC droplets in the liver (Fig. 2A) and the spleen. Liver damage parameters (aspartate transaminase and alanine transaminase) increase transiently [6, 59, 68], although the storage of the inherently inert PFC does not cause organ damage per se, except for the formation of foamy cells from primary Kupffer cells (Fig. 2A) [84, 105]. The increase of plasma transaminase activity seems to be linked to oxidative stress caused by the O_2_ release from the PFC droplet that occurs inside the macrophages (Fig. 2D) [4, 56]. Furthermore, once PFC has reached the liver, it starts to uncouple cytochrome P-450 (CYP450) enzyme reactions. The PFC and the enzyme form a pseudo-enzyme-substrate complex that leads to the hydrolysis of the coenzyme NADPH without substrate conversion. These enzymes are monooxygenases that bind O_2_ to potentially poisonous organic compounds to increase their water solubility for renal clearance [96]. The degree of the enzyme-substrate complex formation depends on the lipophilicity of the PFC and impairs the detoxification capacity of the liver [58, 70, 73]. The NADPH depletion causes the malic enzyme to regenerate NADPH at the expense of malate. Subsequently, less malate transforms into oxaloacetate inside the mitochondria, thus producing less NADH+H^+^. As a result, oxaloacetate and NADH+H^+^ are missing for gluconeogenesis (Fig. 2B) [60]. This forces the liver to deplete glycogen [85]. The liver compensates the detoxification impairment by upregulating the CYP450 biosynthesis [60, 71]. Furthermore, PFCs lead to a dysregulated lipid storage and utilization in the liver (lack of lipogenesis as well as the inhibition of the uptake of chylomicrons and lipoproteins), which might also result from their CYP450 uncoupling (Fig. 2B) [21, 67, 96]. The organ retention can last up to 130 days, while the PFC is constantly excreted via lipoproteins that transport it to the lung, where it evaporates and leaves the body [18]. In between this long period, the PFC seems to be kept from evaporating. Therefore, one might wonder what is happening inside the organs. With increasing lipophilicity the PFCs tend more to interact with lipid membranes [69]. As mentioned above, the PFCs are generally lipophobic, but, e.g., PFOB displays enough lipophilicity to intercalate into the centre of the lipid bilayers of cell membranes up to 2 mol% without causing conformational changes in the membrane (Fig. 2A) [19]. Still, this intercalation is suspected to cause the inhibition of the function of macrophages and neutrophil granulocytes leading to attenuated immune response [72].

## PFC-based oxygen carriers: peculiarities and fields of application

Within the few last years, there has been an important shift of paradigm from large-volume “blood replacement” (several litres) to a small-volume use (mostly 250–500 ml) of artificial oxygen carriers as “oxygen therapeutics” [23]. The major field of application for PFOCs is clearly the short-term blood replacement in cases of blood loss and many reviews already cover this topic [23, 36, 41]. So far, PFOCs can be divided into four subclasses considering the main PFC used in the product: (I) PFD-based PFOCs such as Fluosol-DA, Perftoran and A-AOCs, (II) PFOB-based PFOCs such as Oxygent, (III) tertbutylperfluorocyclohexane-based PFOCs such as Oxycyte and (IV) DDFP-based PFOCs. Their general properties are listed in Table 2. In the USA, the FDA granted the approval for Fluosol-DA in cases of coronary angioplasty but withdrew their approval in 1994 because of its extremely limited shelf life and mainly because of severe complement activation [55]. To treat severe blood loss, Perftoran is approved in Russia, Kazakhstan, Kyrgyzstan and Ukraine and was approved in Mexico from 2005 to 2010 [61]. Oxygent and Oxycyte reached human trials, but by now were not approved by the FDA or elsewhere. Oxycyte successfully completed phase II trials but was abandoned by the sponsor in 2014 due to lack of patient enrolment and economic reasons. Oxygent reached phase III trials, was transiently abandoned because of safety issues that later were disproved to be product-related and is currently under clinical investigation in China [23].

**Table 2:** Main properties of the most promising approaches to artificial blood products [5, 12, 38, 65, 80, 101, 105]

	Formulation	Mean droplet size	Major side effects
Fluosol-DA	14% PFD. 6% perfluorotripropylamine + 2.7% pluronic F-68 + 0.4% egg yolk phospholipid + 0.03% potassium oleate	0.12 μm	Transient drop in neutrophils and platelets, pneumonia [74, 80]
Perftoran	14% PFD. 6% perfluoromethylcyclohexylpiperidin + 6.5% proxanol 268 + egg yolk phospholipid	0.03–0.15 μm	Hypotension and pulmonary complications [61]
Oxygent	58% PFOB. 2% perfluorodecyl bromide + 3.6% egg yolk phospholipid	0.16 μm	Flu-like symptoms, stroke [49]
Oxycyte	60% tertbutylperfluorocyclohexane. Egg yolk phospholipid	0.2 μm	Flu-like symptoms [49]
DDFPe	2% DDFP. 5% human serum albumin	0.2 μm	Coughing, hypertension [12]
A-AOCs	17% PFD. 5% human serum albumin	0.35 μm	Results of clinical studies are not yet available [105]

**Fig. 3:**
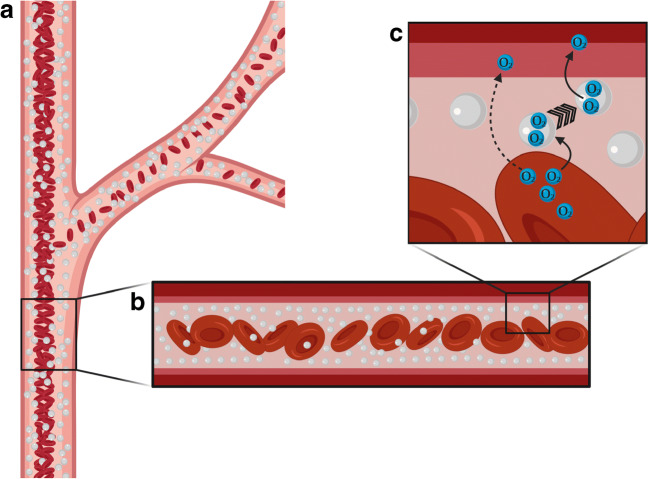
(A) RBCs are located in the middle of the bloodstream, surrounded by a cell-free plasma layer. At bifurcations, the RBC concentration decreases (plasma skimming) [79]. (B) The nano-sized PFC droplets (light grey) are equally distributed in the vessel and do not rely on plasma skimming [39]. (C) The uptake of O_2_ into the PFC droplet happens fast, which shortens the diffusion distance between RBCs and endothelium whilst PFC droplets act as stepping-stones for O_2_ [20]. Figure created with Biorender.com

A closer look at the physiological behaviour of blood in the capillaries in comparison to the properties of PFOCs is worthwhile. Because of the Fåhræus-Lindqvist effect in small vessels, RBCs are located in the middle of the bloodstream, surrounded by a cell-free plasma layer. This effect has two consequences leading to enhanced tissue oxygenation in the presence of PFOCs. The first consequence is the so-called plasma skimming, which occurs at bifurcations of the vessels (Fig. 3A). Whenever a vessel splits into smaller vessels of unequal size, the major fraction of the RBCs remain in the larger vessel, thereby reducing the amount of RBCs within the microcirculation. Subsequently, friction is reduced and the flow in microcapillaries is increased. This is necessary to adjust the O_2_ supply to physiological O_2_ levels in the tissue [79]. However, pathological obstructions exert a similar influence so that the RBC content of the obstructed vessel decreases disproportionally to the flow. Combined with the low haematocrit, this may amplify tissue hypoxia. Moreover, during shock, a narrowing of vessels occurs as the body centralizes through excessive vasoconstriction, again causing a barrier for RBCs. Such vasoconstriction cannot restrain PFOCs from entering and passing partially obstructed vessels, thus maintaining the O_2_ supply for the periphery [13, 39]. Because of the equal distribution of the nano-sized droplets, plasma skimming does not occur with PFC emulsions (Fig. 3B). They maintain the pO_2_ in the microcirculatory system by passing partially obstructed vessels. Recent preclinical and clinical works on heart attack and stroke showed promising results in minimizing the size of the infarct area and, therefore, extending the window for therapy in acute models [3, 11, 12, 22, 92].

The second consequence of the Fåhræus-Lindqvist effect is an increased diffusion distance due to the enlarged cell-free layer surrounding the RBCs [54, 90]. As soon as PFOCs enter the vascular system, they contribute to the O_2_ capacity of the plasma. The uptake of O_2_ into the PFC droplet happens faster than the uptake of O_2_ into the RBCs. This is unimpeded by the emulsifier [54, 106]. Furthermore, PFOCs stay rather close to the endothelia and shorten the diffusion distance between RBCs and the endothelium whilst acting as stepping-stones for O_2_ (Fig. 3C) [20, 37, 78]. This facilitated diffusion is helpful in cases of blood loss caused by extensive bleeding or haemodilution, e. g. during operations assisted by heart-lung machines [103].

However, PFOCs present exceptional physical and physiological properties (Tables 2 and 3), which differ from those of RBCs, which allows them to stand up particularly well in their use in additional pathological conditions.

**Table 3:** Physical and physiological features of PFOCs in comparison to RBCs

	RBCs	PFOCs
Gas transport	Chemically bound	Physically dissolved
Gases	O_2_-, CO_2_-sensitive to oxidative gases	Universal gas dissolubility regardless to oxidative properties
Size	8000–10,000 nm	~ 200 nm (nano-scaled)
Microcirculation	Effected by plasma skimming	Not effected by plasma skimming

One field of application may be the treatment of malignant tumours. Radiotherapy induces the synthesis of reactive oxygen species inside the tumour to cause oxidative stress and finally cell death. Some tumours tend to be metabolically very active so a lack of O_2_ supply and even anoxia occur in the core of the tumour. This anoxia inhibits the formation of reactive oxygen species and renders the tumour cells resistant to radiation therapy [31, 66]. Using PFOCs in this context leads to the oxygenation of the whole tumour, including the core, thereby sensitizing the tumour to radiation therapeutics, even if the patients do not breathe pure O_2_ or carbogen [33, 89, 107].

In the context of organ transplantation, PFC-based oxygen carriers enable the promising new technology of warm machine perfusion. Thereby the actual static cold storage procedures on ice after flushing with crystalline solutions such as University of Wisconsin solution or Custodiol may be replaced in the future. In the last few years, clinical studies showed the advantage of warm machine perfusion after organ harvest to cold storage, either static or perfused [7, 97]. This technique needs O_2_ carriers to maintain the physiological status or to regenerate the tissue. Some new attempts show promising results in the ex vivo perfusion of different organs with PFOCs in low doses oxygenated with carbogen or pure O_2_, respectively [76, 98, 106]. The above-mentioned universal gas dissolubility opens another path off the beaten tracks for PFOCs. The inertness towards oxidation permits the transportation of excess carbon monoxide (CO) from the blood to the lung, and the acceleration of the recovery of CO-venomed haemoglobin [47, 105, 108]. In addition, the washout of nitrogen associated with decompression sickness was proven efficient in terms of avoiding embolism in rabbit, pig and rat models [10, 57, 63, 109]. Severe nitrogen embolism in rats that underwent simulated diving with fast surfacing could be prevented by infusing albumin-derived PFD oxygen carriers prior to diving [64]. However, PFCs do not only wash out poisonous gases but also balance, e.g., local dysregulated nitrogen monoxide (NO) levels. Russian researchers focused on the use of PFC emulsions as a pharmaceutical for directed transport of endogenous bioactive NO to regulate cardiovascular complications associated with NO imbalance [82].

In the 1990s, another field of application for PFC emulsions—use as a contrast agent—was discovered exploiting their ultrasound absorbing properties. DDFP was chosen for this purpose, because of its low boiling point (28 °C), which permits phase transition from the liquid into the gaseous phase, immediately after intravenous injection. The gaseous form results in an extremely short blood half-life of 1.45 ± 0.17 min and a main excretion route via the lung, which minimizes organ retention of DDFP and therefore its negative side effects [2, 12]. These properties of DDFP are in extreme contrast to the other PFCs mentioned above, normally aiming at a rather long intravascular half-life, when PFC emulsions are used as blood substitutes [42]. Nevertheless, DDFPe qualifies as an artificial oxygen carrier therapeutic as the transition leads to a tremendous expansion of intermolecular cavities resulting in an oxygen capacity superior to all other PFCs (Table 1). One application is the short-term oxygenation of hypoxic tissue after stroke [11–13]. Furthermore, DDFP emulsions (DDFPe) can be used for tissue preconditioning prior to a surgery involving tissue ischaemia (e.g. myocard); a technique used to decrease infarct volume during stroke. Here, intravenous administration of DDFPe functions as an oxygen tidal wave resulting in production of reactive oxygen species which is acute but limited in time. These reactive oxygen species subsequently activate the mitochondrial ATP-sensitive K^+^-channel, an effect that exceeds intravascular half-life of DDFPe [11–13, 91].

The current year 2020 presents a major obstacle for humankind in a form of a pandemic that causes thousands to die and millions to be kept in quarantine. The use of PFOCs to treat COVID-19 patients has not yet been tested. However, their use as an additional O_2_ transporter to enhance the peripheral O_2_ supply even in narrowed microcapillaries during severe lung inflammation and their cytoprotective effect on RBCs are already discussed [100].

## Conclusion

Researchers aiming to develop biocompatible artificial oxygen carriers have to work extremely interdisciplinary. More than 50 years after L. C. Clark’s first experiments, researchers worldwide still work in different fields to obtain more and more knowledge on PFOCs. Hopefully, this will lead to safe PFC formulations and allow to exploit these materials to a greater extent as universal gas transporters. The former sprint in the 80s has turned into a marathon because of the complexity of simulating blood. Researchers are still developing new applications and promising new formulations, which make the research field of PFOCs more topical than ever.

## Data Availability

Not applicable.
